# Activating Killer Immunoglobulin Receptors and HLA-C: a successful combination providing HIV-1 control

**DOI:** 10.1038/srep42470

**Published:** 2017-02-13

**Authors:** Mauro S. Malnati, Elisabetta Ugolotti, Maria Cristina Monti, Davide De Battista, Irene Vanni, Domenico Bordo, Francesca Sironi, Patrizia Larghero, Eddi Di Marco, Priscilla Biswas, Guido Poli, Elisa Vicenzi, Agostino Riva, Maciej Tarkowski, Giuseppe Tambussi, Silvia Nozza, Gino Tripodi, Francesco Marras, Andrea De Maria, Angela Pistorio, Roberto Biassoni

**Affiliations:** 1 Unit of Human Virology, Division of Immunology, transplantation and Infectious Diseases IRCCS Ospedale San Raffaele, Milan, Italy; 2 IRCCS Giannina Gaslini, Genoa, Italy; 3 Department of Public Health Unit of biostatistics and clinical epidemiology University of Pavia, Pavia Italy; 4 IRCCS AOU San Martino-IST, Genoa, Italy; 5 Vita-Salute San Raffaele University, School of Medicine, Milan, Italy; 6 Unit of Viral Pathogens and Biosafety, Division of Immunology, Transplantation and Infectious Diseases, IRCCS Ospedale San Raffaele, Milan, Italy; 7 Department of Clinical Sciences Chair of Infectious Diseases and Tropical Medicine University of Milan,“L. Sacco” Hospital, Milan, Italy; 8 Department of Infectious Diseases, IRCCS Ospedale San Raffaele, Milan, Italy; 9 Department of Health Science, DISSAL and Center for excellence in Biomedical Research CEBR University of Genoa, Genoa, Italy

## Abstract

Several studies demonstrated a relevant role of polymorphisms located within the HLA-B and -C loci and the Killer Immunoglobulin Receptors (KIRs) 3DL1 and 3DS1 in controlling HIV-1 replication. KIRs are regulatory receptors expressed at the surface of NK and CD8+ T-cells that specifically bind HLA-A and -B alleles belonging to the Bw4 supratype and all the -C alleles expressing the C1 or C2 supratype. We here disclose a novel signature associated with the Elite Controller but not with the long-term nonprogressor status concerning 2DS activating KIRs and HLA-C2 alleles insensitive to miRNA148a regulation. Overall, our findings support a crucial role of NK cells in the control of HIV-1 viremia.

HIV-1 infection of CD4^+^ T lymphocytes and myeloid cells is characterized by a profound immunodeficiency leading to opportunistic infections and tumor development in most infected individuals in the absence of combination antiretroviral therapy (cART). However, in a minority of people, HIV-1 infection is characterized by either long-term control of their CD4 T cell counts and good health for several years (long-term nonprogressors, LTNP) or spontaneous control of virus replication in peripheral blood (HIV- or Elite-Controllers, EC) for variable periods^1^,^2^. Among the best characterized correlates of these rare (1–2% of infected individuals) conditions of natural control of HIV-1 disease progression, there are genetic factors, mostly associated to the HLA Class I loci, in addition to the heterozygous 32-bp deletion of CCR5 typical of a significant fraction of LTNP^3^. These findings have been largely interpreted and correlated with a robust CD8^+^ cytotoxic T cell (CTL) response keeping in check the number of infected cells^4^. In this regard, CTL act through recognition and killing of infected cells mediated by specific viral peptides presented by selected Class I molecules, mostly belonging to the HLA-B locus^4^. Moreover, evidence has emerged on the epistatic association between the HLA-B supratype Bw4 and the Killer Immunoglobulin–like receptors (KIRs) 3DL1/DS1 locus in the beneficial responses to HIV-1^5^,^6^. Indeed, both innate and adaptive cellular immunity share HLA class I ligands^7^,^8^ and the KIR multigene family^9^,^10^ as fine regulators of their activity.

KIRs are encoded in humans by at least 15 distinct polymorphic gene loci located in a region of 150–200 kb of the leukocyte receptor complex (LRC) on human chromosome 19q13.4^7^,^11^ and display either inhibitory (L) or activating (S) functions^10^,^12^. They are inherited as different haplotypes, resulting in a centromeric and a telomeric KIR profile that varies in number and composition of genes^12^,^13^. The majority of these receptors bind to different groups of HLA class I molecules^14^ encoded by genes located on chromosome 6. Interestingly, evolution drove at least 5 KIRs to recognize HLA-C alleles, the most recently developed forms amongst HLA molecules^15^.

The involvement of other HLA loci, mainly HLA-C, in HIV-1 control has been long questioned^1^,^16^, being dismissed as a simple carryover of the existing linkage disequilibrium (LD) with some protective HLA-B alleles, such as B57:01^17^. Only in recent years, several genetic and functional experimental studies strongly supported an independent involvement of HLA-C molecules in HIV-1 infection outcomes^18^,^19^. Studies based on genome wide association analyses (GWAS) proved that a genetic variant rs9264942 C/T located 35 Kb upstream of the HLA-C locus was associated with control of viremia^20^,^21^, slower disease progression^22^, and the levels of HLA-C cell surface expression in caucasoid populations^23^. Subsequently, the causal variant responsible for these associations was identified as a Single Nucleotide Polymorphism (SNP) (rs67384697G/del) that maps in the 3′ UTR of HLA-C alleles, and affects the binding of the microRNA Hsa-miR-148a^24^. This SNP was shown to partially influence cell surface expression of HLA-C with weakly expressed alleles possessing an intact miR148a binding site (263 G) and highly expressed alleles having a deletion in the site (263del), thus escaping the regulation by that microRNA^24^.

Independent evidence for a causal effect of HLA-C expression on HIV-1 control was provided by the direct relation between the frequency of escape mutations in predicted HLA-C epitopes and viral load^25^,^26^ and by the strong positive correlation between the frequency of HLA-C peptide-restricted CTL responses and HLA-C expression^26^. Noteworthy, HLA-C molecules insensitive to the HIV-1 escape strategy mediated by the viral protein nef can be downmodulated by some variants of the VPu accessory molecules^1^,^27^,^28^.

In this study, we analyzed chromosome 6 genetic markers in combination with the assessment of the KIR genotype in a well characterized cohort of Italian EC and LTNP. Our results provide evidence that a complex genotype displaying high expression of HLA-C alleles belonging to the C2 group and their KIR 2DS receptors ligands is a hallmark of the EC but not of the LTNP status. These findings suggest a novel role for activating 2DS KIRs expressed on both NK and CD8+ T-cells in the control of HIV-1 viremia.

## Results

### The HLA Class I region and KIR genes influence the natural course of HIV-1 infection

**Figure 1:**
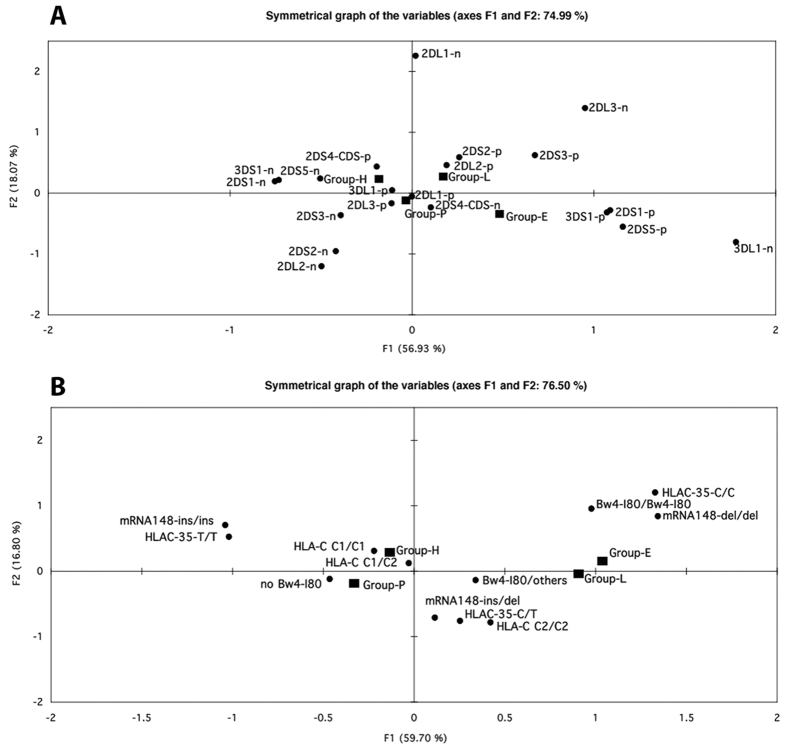
Principal component analysis of genetic markers on chromosome 19 (**A**) or 6 (**B**). Distinct groups of HIV-1 infected individuals and HIV-1 negative donors are indicated with a black filled box (E: Elite Controller, L: Long Term Non Progressor, P: Progressor, H: Healthy HIV-1 negative donors). (**A**) KIR genotypes are shown by black filled circles (p: positive, n: negative). (**B**) HLA-C, HLA-Bw4-I80, −35 SNPs and miRNA148a SNPs homozygous and- heterozygous genotypes are indicated.

An explorative multiple correspondence analysis of selected chromosome 6 polymorphisms within the HLA-B and -C class I region and of KIR genes was conducted independently to verify whether combinations of the selected chromosome 6 and 19 markers could discriminate between the two best characterized cohorts of natural controller of HIV-1 infection, i.e. EC and LTNP, in comparison to HIV-1 Progressors (P) or HIV-1 seronegative healthy blood donors (HD). The presence of activating KIR genes, namely 3DS1, 2DS1 and 2DS5 on chromosome 19 (Fig. 1A) clearly distinguished the 40 EC (lower right quadrant) from the 111 HD (upper left quadrant) and to some extent from the189 HIV P (lower left quadrant), whereas the 39 LTNP segregated in an intermediate position between EC and P.

Both EC and LTNP segregated independently from P and HD for chromosome 6-associated markers (Fig. 1B), that included homozygosity of SNP rs9264942C, commonly termed −35C, and of SNP rs67384697del (263del) as well as the presence of HLA-B alleles both belonging to the Bw4-I80 supratype.

As both groups of HIV-controllers segregated in close proximity they were also jointly analysed and compared with P (Supplemental Fig. 1), confirming the strong association with the homozygous status for both HLA-C-associated SNPs and the presence of Bw4-I80 alleles. As expected, HD segregated with P (Fig. 1A,B), the latter representing the natural evolution of HIV infection in most (≥95%) individuals.

### Distinct sets of KIRs and HLA genes are involved in HIV-1 disease control

**Figure 2:**
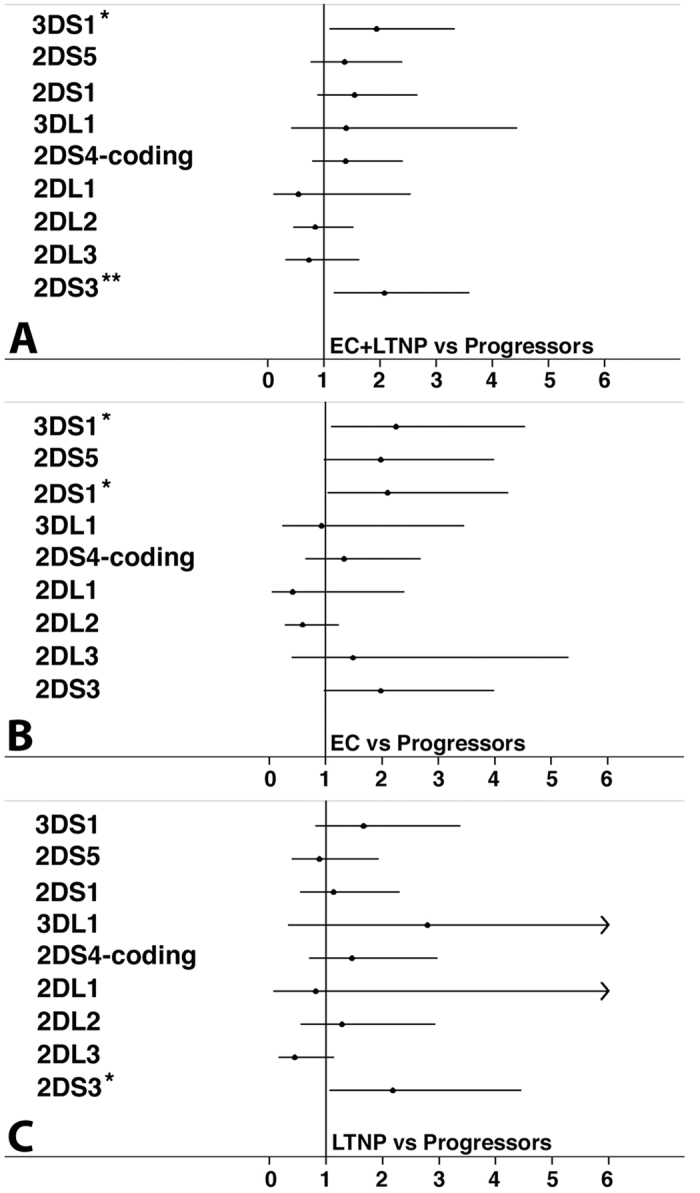
Effect of chromosome 19 genetic polymorphisms on lack of HIV disease progression. The estimate of the risk was calculated comparing EC and/or LTNP vs HIV-1+ P. Panels (A–C) show the odds ratio and 95% confidence interval considering an additive allelic genetic model. The asterisk indicates bonferroni corrected significant p-values: *p < 0.05, **p < 0.01.

The study of the univariate associations (Fig. 2A) between the status of natural HIV-1 controllers, encompassing both EC and LTNP, and KIR genes confirmed a role for the 3DS1 gene (p < 0.05), but also showed a strong association (p < 0.01) with another activating KIRs, the 2DS3 gene. Interestingly, when the two groups of controllers were independently analysed and compared with P (Fig. 2B,C), EC showed a different behaviour from LTNP as suggested by the multiple correspondence analysis. The telomeric B haplotype activating KIRs 3DS1 and 2DS1 increased the odds of being an EC (OR = 2.25, p = 0.02 and OR = 2.10, p = 0.03, respectively), whereas the other 2DS3/5 activating KIRs were of borderline statistical significance (OR = 1.98, p = 0.055) (Fig. 2B). Conversely, only the 2DS3 gene was significantly associated with the LTNP status (OR = 2.2, p = 0.03) (Fig. 2C).

**Figure 3:**
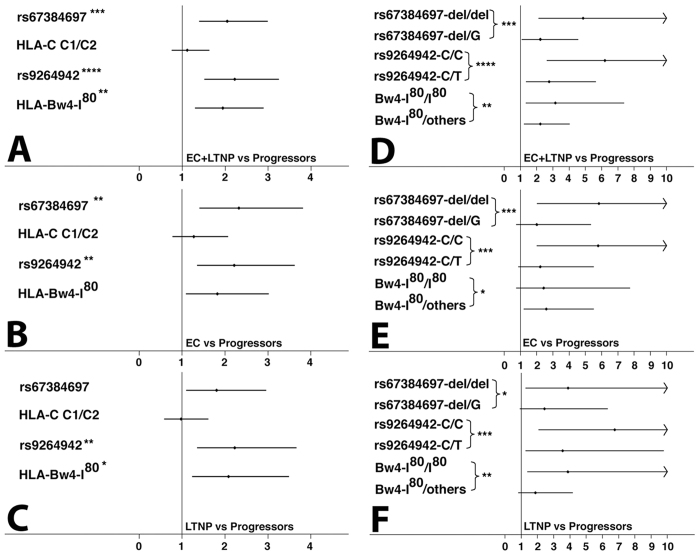
Effect of chromosome 6 genetic polymorphisms on HIV control. The estimate of the risk was calculated comparing EC and/or LTNP vs HIV-1 + P. Panels (A–C) show the odds ratio and 95% confidence interval considering an additive allelic genetic model. The asterisk indicates bonferroni corrected significant p-values (*p < 0.05, **p < 0.01, ***p < 0.001, ****p < 0.0001). Panels (D–F) show the odds ratio and 95% confidence intervals considering a genotypic genetic model (presence of two copies or one copy of the risk allele vs no copies). The asterisk indicate chi-square test for trend p-value for the statistically significant polymorphism:(*p < 0.05, **p < 0.01, ***p < 0.001, ****p < 0.0001).

We next investigated the allelic association between HLA genes and the HIV-1controller status (Fig. 3). Both HLA-C-associated SNPs and the Bw4-I80 supratype were significantly associated with elite controller status, while the HLA C1/C2 supratype was not associated (Fig. 3A). Of note, EC and LTNP were characterized by an inverse pattern of the 263del and the Bw4-I80 supratype, the former being the strongest marker of the EC status and the latter the weakest with borderline significance (Fig. 3B), whereas this pattern was switched in LTNP (Fig. 3C). In addition, the effect of allele dosage for these three significant markers was investigated (Fig. 3D–F). Homozygosity for 263del, −35 C and the presence of two HLA-B alleles carrying the Bw4-I80 epitope significantly increased the odds of being a HIV-1 controller compared to heterozygosity (Fig. 3D; p for trend <0.01 or less). Of interest, both HLA-C-associated SNPs showed the best association (−35 C/C OR = 6.2, CI = 2.6–14.6; 263 del/del OR = 4.9, CI = 2.1–11.1), whereas the effect of Bw4 I80/I80 was weaker (OR = 3.1, CI = 1.3–7.4).

A difference was noted again between ECs and LTNPs (Fig. 3E,F): the allele dosage effect of Bw4-I80 was observed only in LTNPs where the odds conferred by Bw4-I80/I80 were higher than those associated with the Bw4-I80/others genotypes (OR = 3.9, CI = 1.4–10.9, OR = 1.9, CI = 0.85–4.2, respectively, p for trend <0.01).

### HIV-controllers are characterized by a complex chromosome 6 haplotype

We tested whether the overall haplotype variation among the markers selected on chromosome 6 influenced the HIV-1 controller phenotype. As HLA genes are located at adjacent loci and are presumed to exhibit epistasis with each other or with other genes, we assessed the degree of LD in HD (see Material and Methods) matched for ethnicity and geographical origin with our HIV-1 infected groups. Only the HLA-C-related SNPs (Supplemental Table 1) showed a moderate degree of LD (D’ = 0.62). The haplotype containing the three significant alleles in the univariate analyses (263del, −35C, Bw4-I80) clearly increased the odds of being either EC (OR = 3.5, p = 0.0013) or LTNPs (OR = 3.5, p = 0.0025) (Table 1).

Surprisingly, the addition of the C2 supratype strongly reinforced the haplotypic association with the status of EC (OR = 6.1, p = 0.00167) but not of LTNP (OR = 3.9, p = 0.0136). In contrast, a haplotype containing the C1 marker was neither significantly associated with the status of ECs nor of LTNPs. These findings suggest that the presence of the HLA-C2 alleles carrying a miRNA 148a/b deleted site may play a key role in determining the EC status.

### EC are identified by a unique genetic configuration

We assessed the distribution of each significant KIR gene in HIV-1 controllers and P (Table 2), conditioning on the presence/absence of the described four marker-haplotype (Haplo4+/Haplo4−) on chromosome 6. Among Haplo4+ carriers only haplotype B activating KIRs (3DS1, 2DS5, 2DS1, 2DS3) were significantly associated with the EC status. We extended the previously described association between 3DS1 and the presence of the Bw4-I80 allele^5^ to ECHaplo4+ carriers, but, more importantly we described an even stronger association between activating 2DS genes and the haplotype featuring C2 alleles insensitive to the miRNA 148a/b regulation that could be considered a hallmark of EC (Table 2). In contrast, LTNP did not show a relevant association with any of the activating KIRs beside a modest association with the B centromeric 2DS3 gene in the the Haplo4− individuals (Table 2).

**Figure 4:**
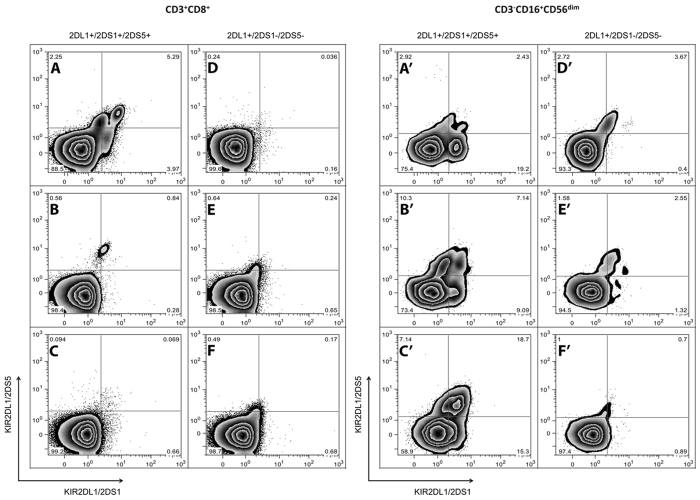
Surface expression of KIR 2DS1 and 2DS5 on circulating CD8 and NK-cells. Resting PBMCs are gated according to anti-CD3 and anti-CD8 mAbs (CD3+ CD8+ T lymphocytes: panels A–F) or anti-CD3, anti-CD16 and anti-CD56 mAbs (CD3-,CD16+ CD56dim NK lymphocytes: panels A’–F’). Zebra plots of PBMCs stained with MAbs reacting only with the 2DL1/2DS5 (y axes) or with the 2DL1/2DS1 molecules (x axes) in EC positive (panels A–C and A’–C’) or negative (panels D-F and D’-F’) for the 2DS1 and 2DS5 gene are shown.

Finally, the expression of the activating 2DS1 and 2DS5 molecules was assessed by cell surface immunostaining of circulating resting CD3+CD8+ T lymphocytes (Fig. 4A–F) and CD3-CD16+ CD56dim NK cells (Fig. 4A’–F’) in six EC. All three ECs characterized by the 2DL1+ 2DS1+ 2DS5+ genotype presented both 2DS1 and 2DS5 expressing NK populations (Fig. 4A’–F’) which were undetectable in the three EC carrying the 2DL1+ 2DS1–2DS5- genotype (Fig. 4D’–F’). Only one individual carrying the 2DL1+ 2DS1+ 2DS5+ genotype presented 2DS1 and 2DS5 positive populations within CD8+ T lymphocytes (Fig. 4A).

## Discussion

This paper describes an in-depth combined analysis of chromosome 6 genetic markers with the complete KIR genotype configuration revealing, for the first time, a crucial role of 2DS KIR receptors and their HLA-C2 ligands, insensitive to miRNA148a regulation, in the control of HIV viremia naturally occurring in EC of caucasoid origin.

By exploiting two different groups of HIV-1 controllers, namely 40 EC and 39 LTNP with persistent and detectable plasma viral load, we revealed a distinct role of Bw4-I80 HLA-B expressing alleles and miRNA148a insensitive HLA-C alleles in HIV-1 control, pin-pointing a novel association of the 2DS KIRs with the EC status.

All the statistical approaches conducted separately on both groups of HIV-controllers indicated substantial differences between EC and LTNP. Indeed epidemiological studies conducted on large populations of HIV-1 infected individuals^29^ indicated that these two phenotypes of HIV-1 controllers did not overlap, with extremely few individuals showing features of both EC and LTNP. However, the genetic or immunologic correlates distinguishing LTNP and EC have remained elusive thus far.

The multiple correspondence and logistic regression analyses performed on the KIR genotyping data set indicated a significant association between the 3DS and 2DS loci and EC. Interestingly, the cumulative analysis of both groups as HIV-controllers confirmed a role only for the KIR3DS1 locus, as previously described^5^.

Furthermore, consistent differences were found between the effects of HLA-C associated SNPs and the Bw4-I80 supratype. Of interest, opposite effects are shown by the *263del* and the *Bw4-I80* polymorphisms in EC and LTNP. Indeed, the analysis of the allele dosage suggested a clear advantage of *263del* homozygosity in defining the EC status, whereas the presence of both HLA-B alleles carrying the *Bw4-I80* supratype was not significant. In contrast, the presence of both HLA-*Bw4-I80* expressing alleles was advantageous for LTNP, whereas homozygosity for *263del* is the least powerful marker in defining the LTNP status.

These observations underscored a major role of HLA-C alleles escaping miRNA 148a regulation in EC and a primary role of HLA-B alleles carrying the *Bw4-I80* epitope in LTNP. Intriguingly, the *-35 C/C* polymorphism identified by GWAS studies^20^,^21^ was revealed to be the strongest predictor either for HIV-controllers or LTNP status. Taken together, these findings suggest that the *-35* SNP could serve as an associated marker for both the *263del* and HLA-B allele belonging to the *Bw4-I80* supratype. Thus, consistent with its chromosome 6 location between the HLA-B and -C loci, the *-35* SNP may flag the HLA-B and -C driven T-cell response of both LTNP and EC^1^,^30^ whereas additional mechanisms for HIV-control linked to HLA-C expression are likely present only in EC.

The presence of multiple immune mediated mechanisms responsible for HIV-control in EC and LTNP is further underlined using haplotypic models to test interactions of chromosome 6-associated markers in defining the EC or LTNP status. The increasing odds ratios obtained by adding progressively each significant marker (*-35 C/C, 263del* and *Bw4-I80*) underlines the presence of at least one shared mechanism responsible for HIV-control. On the other hand, the strong effect caused by the addition of the C2, but not of the C1 supratype, indicates an independent contribution of C2 alleles escaping miRNA148a regulation in defining the EC, but not the LTNP, linking, for the first time, this HLA-C-related peculiarity to HIV-control.

Finally, the stratified analysis that combined activating KIR loci and the best protective chromosome 6 haplotype (Haplo 4) confirmed a role of both the 3DS1/Bw4-I80 and 2DS/C2 263del pairs in defining again the EC status. Thus, only EC display a genotype suggestive for epistasis between activating KIRs and their best HLA-B and -C2 ligands. Therefore, NK cells of individuals displaying a complete KIR B haplotype, including both 3DS1 and multiple 2DS genes^13^ and their specific HLA-B and -C ligands, might be facilitated in overcoming KIR-mediated inhibitory signals, thus contributing to direct recognition and faster clearance of HIV-infected cells. Indeed, the 2DS1 receptor^31^,^32^ and, more recently, the 3DS1 molecule^33^ were shown to bind HLA-C2 and HLA-Bw4-I80 alleles, respectively, with a weak interaction. In this regard, the selective association with the C2 alleles displaying a higher surface expression^14^,^24^ may compensate for the lower binding affinity of the 2DS1 molecule. In addition, the binding of 3DS1 to the HLAB-57:01 molecule (a Bw4-I80 allele) is strongly influenced by the type of peptide loaded in the MHC binding cleft^33^ supporting a model whereby changes in the peptide repertoire due to viral infection trigger the activating KIR engagement and NK-cell mediated elimination of infected cells. Although, the binding of HLA-C alleles has never been demonstrated for the 2DS3/5 receptors^32^, a similar peptide dependence could be hypothesized also for these receptors which are closely related to the HLA-C2 specific 2DL1/S1 molecules (Supplemental Figure 2A). Notably, the neighbour-joining tree representing the similarity of the KIRs amino acidic residues involved in the HLA-C/peptide interaction according to the known crystallographic structures showed that the 2DS3/5 receptors are the closest molecules to the 2DL1/2DS1 receptors (Supplemental Figure 2B).

Indeed, these two KIR molecules share 17/18 of the amino acidic residues present in the interface between HLA-C and 2DL molecules. Noteworthy, the unique amino acidic change (M44T) featured by both 2DS3 and 2DS5 is compatible (Supplemental Figure 3) with the retainment of HLA-C binding involving the side chains of neighbouring amino acids (Arg 79 and/or Lys 80) in hydrogen bond interactions with the Thr 44 side chain. Along this line, replacement of the Met 44 with Thr in 2DL1*003 molecules does not abrogate C2 specificity retaining 50% of avidity^34^. Our findings support the view that a functional combination of a complex HLA-B and -C haplotype with a KIR B haplotype containing a full array of activating KIRs represents a major mechanism of HIV-control in EC, as it is shared by almost 40% of individuals. Thus, it is likely that HLA-C molecules may participate directly in the immune response against HIV-1 triggered by activating KIR mediated NK-cell responses and a CD8 T-cell selective pressure on the virus. This association was independent from the time-span of HIV-seropositivity and whether the infection occurred before or after the introduction of cART. Unfortunately, given the Italian origins of our cohort of HIV-controllers it was not possible to evaluate whether this complex genotype is featured also by Sub-saharian African populations, known to be characterized by different allelic forms and distribution of activating KIRs genes^35^.

Notably, the present data show that in EC the presence of an activating KIR genotype correspond to their cell surface expression mainly on NK cells, supporting a predominantly direct NK-cell mediated mechanism of HIV control, with downstream editing of CD8+ T cell responses.

Although the limited number of individual tested for KIR expression does not allow us to draw a definitive conclusion, these findings suggest a crucial involvement of NK cells in the immune control of HIV-1-viremia through a network of coexpressed 2DS/3DS1 molecules. These data are in line with the distinctive persistence of effective induction of activating natural citotoxicity receptors in the vast majority of HIV-controllers^36^.

The engagement of both NCRs and, more importantly, the 2DS/3DS1 KIRs receptors in individuals carrying a complex HLA haplotype that combines both HLA-Bw4-I80 and C2 alleles escaping miRNA 148a regulation will be crucial in mounting a quick antiviral response that limits and maintain under control the viral reservoire of infected cells.

## Methods

### Patients

Genomic DNA was extracted from circulating mononuclear cells derived from a group of 79 HIV–controllers belonging to Caucasoid ethnicity encompassing 40 Elite Controllers (EC) featuring one or more yrs of HIV–1 plasma RNA load <50 copies/ml (median = 14; 1–3° IQR = 10–21) and 39 Long Term Non Progressors (LTNP) with a documented history of HIV–1 seropositivity >9 yrs (median = 17; 1–3° IQR = 13–22). A control group of 189 HIV–1 Progressors (P) included 93 individuals with immunological reconstitution following cART, 61 ART–naïve viremic individuals with a documented history of HIV–seropositivity <7 yrs (median = 3; 1–3° IQR = 1–6) requiring the introduction of cART and 35 individuals with primary HIV-infections or recent seroconverters.

Finally, genomic DNA samples derived from 111 HIV–seronegative healthy blood donors were utilized for conducting only the multiple components and the LD analysis. Demographic and laboratory features of these populations are summarized in Supplemental Table 1.

### KIR genotyping, HLA and SNPs genotyping

KIR genotyping was performed by a modification of a published SSP–PCR system^37^. HLA supratyping used a published pyrosequencing approach^38^,^39^,^40^ that relies on a nested PCR protocol where the first amplification with a separate set of primers amplifies all HLA-B or HLA-C alleles, whereas a second PCR reaction is used to amplify the fragment subjected to pyrosequencing, distinguishing Bw4 (Iso/Thr)^80^ from Bw6 HLA–B alleles and Ser^77^/Asn^80^ or Asn^77^/Lys^80^ HLA–C alleles representing C1 or C2 KIR ligands, respectively. The HLA supratyping data were independently verified using classical HLA typing approaches in 50% of the cases with a particular attention to samples that resulted identical for HLA-B and -C supratyping^38^.

The single nucleotide polymorphism rs9264942 C/T (-35) has been analyzed using quantitative pyrosequencing selecting primers outside of the polymorphic regions. Sequencing has been performed with the 5′TCA GAA AGT CCC ACA GT3′ oligonucleotide following the nucleotide dispensation order: TGCT[A/G]CAG and polymorphism is evaluated in the anti-sense orientation. The same method was adopted to analyze the rs67384697 (G/deletion) polymorphism centered at the residue–263 of the 3′UTR region of HLA–C alleles^41^. SNPs analysis of homozygous samples was repeated at least twice.

### Monoclonal Antibodies (mAb) and cell surface staining

Anti–CD8 (clone SFCI21Thy2D3) and anti–CD3 (clone SK7) mAbs were purchased from Beckman Coulter; anti–CD16 (clone 3G8) and anti–CD56 (clone B159) mAbs were from Becton Dickinson. The KIR receptors 2DL1/2DS1 were detected using the mAb 11PB6^42^ (MiltenyiBiotec), whereas mAb 143211 (R&D Systems) was used to detect the 2DL1/2DS5 molecules^43^. LIVE/DEAD fixable Aqua dead cell stain kit was purchased from Invitrogen.

The samples were acquired with a Gallios flow cytometer (Beckman Coulter, Inc.) and analyzed using FlowJo version 8.8.7 (Tree Star). Lymphocytes were gated on a forward scatter versus side scatter area using pseudo-colour dot plot and dead cells were removed according to positive Aqua stain. CD8^+^ T–cells were identified within the CD3^+^ -lymphocytes, whereas NK cells (CD16^+^ CD56dim) were gated within the CD3^−^ cells. Cells stained by the KIR–specific mAbs binding KIR2DL1/2DS1 (mAb 11PB6) and KIR2DL1/2DS5 (MAb 143211) were plotted on the x–axis and y–axis respectively, allowing the discrimination between 2DL1, 2DS1 and 2DS5 KIR positive cells.

### Statistics

A power analysis was employed to calculate the dimension of the HIV-controllers and Progressors groups. Four HIV progressors were excluded from the analysis due to insufficient sample material that did not allow the complete genetic marker testing. The statistical power of 80% was achieved for searching allelic Odds Ratios (OR) ≥2, considering a type 1 error level of 5% and minor allele frequencies (MAF) between 20% and 40% of the above described groups of HIV–1 infected individuals. To assess the association between each genetic marker and HIV–controller status (EC and/or LTNP) we performed univariate allelic Pearson chi–square or Fisher exact tests when appropriate and applied Bonferroni correction considering the number of markers tested for chromosome 6 and 19; moreover, the Cochran–Armitage trend test was applied to evaluate a possible additive dose-response effect of the considered risk alleles of chromosome 6. Adjusted Odds Ratio (OR) with 95% confidence intervals (95% CI) were derived and used as the measure of effect.

The global association of the EC or LTNP status with the significant chromosome 6 or 19 genetic markers was explored by multiple correspondence analysis^44^. To test whether the overall haplotype variation of the chromosome 6 HLA markers influences the HIV–controller status, we performed a Likelihood Ratio (LR) based omnibus association test, considering specific haplotypes. In addition, we tested whether a single marker had an independent effect (independent of the haplotypic effects formed by the remaining markers) or it was associated to EC and/or LTNP status due to Linkage Disequilibrium (LD), performing conditional LR–based tests and asymptotic p-values. To take in account the possible role of a chromosome 6 risk haplotype, we carried out a stratified analysis on each chromosome 19 significant marker. The Mantel–Haenszel method provided pooled and separated odds ratio for each strata (presence/absence of the chromosome 6 haplotype). The allele frequencies in controls were examined to detect any significant deviation from the Hardy–Weinberg Equilibrium (HWE) using a goodness of fit Chi–square test. LD was calculated using HAPLOVIEW software. Statistical analyses were performed using Plink 1.08^45^, XLstat (Version 2, Addinsoft) and Stata 11 (Stata Corporation, College Station, TX, USA).

### Study approval

All the experiments were performed in accordance with relevant guidelines and regulations and all participants gave a written informed consent to the study protocol prior to inclusion in the study. This study was approved by the IRCCS AOU San Martino-IST Genoa n51/09, ALS 2 n°10/2011, IRCCS San Raffaele Hospital, Milan (EUDRACT numbers 2003-P-001678/14; 2008-006287-11;2008-007004-29) and by L Sacco Hospital Milan (ELVIS 2008/419) ethical Commitees.

## Additional Information

**How to cite this article**: Malnati, M. S. *et al*. Activating Killer Immunoglobulin Receptors and HLA-C: a successful combination providing HIV-1 control. *Sci. Rep.* **7**, 42470; doi: 10.1038/srep42470 (2017).

**Publisher's note:** Springer Nature remains neutral with regard to jurisdictional claims in published maps and institutional affiliations.

## Supplementary Material

Supplementary Materials

## Figures and Tables

**Table 1:** Haplotypic combination of chromosome 6 markers in different populations of HIV-controllers and Progressors.

Haplotypiccombination	EC (N = 40) versus Progressor (N = 185)		LTNP (N = 39) versus Progressor (N = 185)	
	OR (95% CI)	P	OR (95% CI)	P
rs67384697 and rs9264642	2.8 (1.6–4.9)	0.00024	2.5 (1.4–4.5)	0.00131
rs67384697 and Bw4–I^80^	2.4 (1.3–4.3)	0.00546	2.3 (1.2–4.2)	0.00938
rs9264642 and Bw4–I^80^	3.1 (1.6–6.1)	0.00027	2.8 (1.4–5.6)	0.00315
rs67384697, rs9264642 and Bw4–I^80^	3.5 (1.6–7.4)	0.00130	3.5 (1.6–7.9)	0.00246
rs67384697, rs9264642, Bw4–I^80^ and HLA–C2	6.1 (2.0–18.1)	0.00167	3.9 (1.3–11.4)	0.0136
rs67384697, rs9264642, Bw4–I^80^ and HLA–C1	2.5 (0.8–7.6)	0.107	2.8 (0.9–8.9)	0.083

**Table 2:** Logistic multivariate analysis of KIRs in EC or LTNP individuals versus Progressors, in the presence (Haplo4+) or absence (Haplo4−) of the chromosome 6 aplotype.

KIRs	EC (N = 40) versus Progressor (N = 185)		LTNPs (N = 39) versus Progressor (N = 185)	
	OR_MH_(95% CI)	*P*^*§*^	OR_MH_(95% CI)	*P*^*§*^
3DS1				
Haplo4+	6.82 (1.56–29.87)	**0.0031**	2.25 (0.55–9.13)	0.2436
Haplo4−	1.36 (0.56–3.35)	0.4966	1.74 (0.74–4.09)	0.2019
2DS5				
Haplo4+	4.79 (1.18–19.42)	**0.0153**	1.15 (0.23–5.65)	0.8633
Haplo4−	1.38 (0.55–3.46)	0.4846	0.94 (0.38–2.33)	0.8957
2DS1				
Haplo4+	6.82 (1.56–29.87)	**0.0031**	1.64 (0.40–6.70)	0.4862
Haplo4−	1.26 (0.51–3.09)	0.6116	1.16 (0.50–2.72)	0.7257
2DS4-CDS				
Haplo4+	3.14 (0.84–11.72)	0.0719	0.94 (0.20–4.51)	0.9412
Haplo4−	0.94 (0.37–2.38)	0.8952	2.09 (0.88–4.96)	0.0863
2DS3				
Haplo4+	7.67 (1.69–34.82)	**0.0018**	2.40 (0.55–10.50)	0.2315
Haplo4−	0.93 (0.36–2.44)	0.8879	2.55 (1.07–6.08)	0.0293

OR_MH_: Mantel–Haenszel Odds Ratio; ^***§***^P:P value refers to the Mantel–Haenszel Chi–square test.

## References

[b1] Deeks S. G. & Walker B. D. Human immunodeficiency virus controllers: mechanism of durable virus control in the absence of antiretroviral therapy. Immunity 27, 406–416 (2007). 17892849 10.1016/j.immuni.2007.08.010

[b2] Riva A., Vicenzi E., Galli M. & Poli G. Strenuous resistance to natural Hiv-1 disease progression: viral controllers and long-term nonprogressors. Future Virology 6, 521–533 (2011).

[b3] Carrington M., Dean M., Martin M. P. & O’Brien S. J. Genetics of HIV-1 infection: chemokine receptor CCR5 polymorphism and its consequences. Hum. Mol. Genet. 8, 1939–1945 (1999). 10469847 10.1093/hmg/8.10.1939

[b4] Goulder P. J. & Walker B. D. HIV and HLA class I: an evolving relationship. Immunity 37, 426–440 (2012). 22999948 10.1016/j.immuni.2012.09.005 PMC3966573

[b5] Martin M. P. . Epistatic interaction between KIR3DS1 and HLA-B delays the progression to AIDS. Nat. Genet. 31, 429–434 (2002). 12134147 10.1038/ng934

[b6] Martin M. P. . Innate partnership of HLA-B and KIR3DL1 subtypes against HIV-1. Nat. Genet. 39, 733–740 (2007). 17496894 10.1038/ng2035 PMC4135476

[b7] Wende H., Colonna M., Ziegler A. & Volz A. Organization of the leukocyte receptor cluster (LRC) on human chromosome 19q13.4. Mamm. Genome 10, 154–160 (1999). 9922396 10.1007/s003359900961

[b8] André P. . New nomenclature for MHC receptors. Nat. Immunol. 2, 661 (2001). 11477395 10.1038/90589

[b9] Hsu K. C. . Killer Ig-like receptor haplotype analysis by gene content: evidence for genomic diversity with a minimum of six basic framework haplotypes, each with multiple subsets. J. Immunol. 169, 5118–5129 (2002). 12391228 10.4049/jimmunol.169.9.5118

[b10] Biassoni R. Human Natural Killer Receptors, Co-Receptors, and Their Ligands. Curr. Prot. Immunol. Unit 14.10 (Wiley, J. & sons Eds, 2009). 10.1002/0471142735.im1410s84 19235767

[b11] Biassoni R., Ugolotti E. & De Maria A. Comparative analysis of NK cell receptor expression and function across primate species: perspective on antiviral defenses. Self/Nonself 1, 103–113 (2010). 21487512 10.4161/self.1.2.11717 PMC3065668

[b12] Middleton D. & Faviel G. The extensive polymorphism of KIR genes. Immunol. 129, 8–19 (2009). 10.1111/j.1365-2567.2009.03208.x PMC2807482 20028428

[b13] Pyo C. . Different patterns of evolution in the centromeric and telomeric regions of group A and B haplotypes of the human killer cell Ig-like receptor locus. PLoS ONE 5, e15115 (2010). 21206914 10.1371/journal.pone.0015115 PMC3012066

[b14] Martin M. P. & Carrington M. Immunogenetics of HIV disease. Immunol. Rev. 254, 245–264 (2013). 23772624 10.1111/imr.12071 PMC3703621

[b15] Parham P., Norman P. J., Abi-Rached L. & Guethlein L. A. Human-specific evolution of killer cell immunoglobulin-like receptor recognition of major histocompatibility complex class I molecules. Philos. Trans. R. Soc. Lond. B. Biol. Sci. 367, 800–811 (2012). 22312047 10.1098/rstb.2011.0266 PMC3267113

[b16] Corrah T. W. . Reappraisal of the relationship between the HIV-1-protective single-nucleotide polymorphism 35 kilobases upstream of the HLA-C gene and surface HLA-C expression. J. Virol. 85, 3367–3374 (2011). 21248048 10.1128/JVI.02276-10 PMC3067890

[b17] Kiepiela P. . Dominant influence of HLA-B in mediating the potential co-evolution of HIV and HLA. Nature 432, 769–775 (2004). 15592417 10.1038/nature03113

[b18] Kulpa D. A. & Collins K. L. The emerging role of HLA-C in HIV-1 infection. Immunology 134, 116–122 (2011). 21896007 10.1111/j.1365-2567.2011.03474.x PMC3194220

[b19] Zipeto D. & Beretta A. HLA-C and HIV-1: friends or foes? Retrovirology 9, 39–46 (2012). 22571741 10.1186/1742-4690-9-39 PMC3386009

[b20] Fellay J. . A whole-genome association study of major determinants for host control of HIV-1. Science 317, 944–947 (2007). 17641165 10.1126/science.1143767 PMC1991296

[b21] Pereyra F. and the International HIV Controllers Study. The major genetic determinants of HIV-1 control affect HLA class I peptide presentation. Science 330, 1551–1557 (2010). 21051598 10.1126/science.1195271 PMC3235490

[b22] Fellay J. .Common genetic variation and the control of HIV-1 in humans. PloS Genet. 5, e1000791 (2009). 20041166 10.1371/journal.pgen.1000791 PMC2791220

[b23] Thomas R. . HLA-C cell surface expression and control of HIV/AIDS correlate with a variant upstream of HLA-C. Nat. Genet. 41, 1290–1294 (2009). 19935663 10.1038/ng.486 PMC2887091

[b24] Kulkarni S. . Differential microRNA regulation of HLA-C expression and its association with HIV control. Nature 472, 495–498 (2011). 21499264 10.1038/nature09914 PMC3084326

[b25] Blais M. E. . High frequency of HIV mutations associated with HLA-C suggests enhanced HLA-C-restricted CTL selective pressure associated with an AIDS-protective polymorphism. J. Immunol. 188, 4663–4670 (2012). 22474021 10.4049/jimmunol.1103472 PMC3378658

[b26] Apps R. . Influence of HLA-C expression level on HIV control. Science 340, 87–91 (2013). 23559252 10.1126/science.1232685 PMC3784322

[b27] Collins K. L., Chen B. K., Kalams S. A., Walker B. D. & Baltimore D. HIV-1 Nef protein protects infected primary cells against killing by cytotoxic T lymphocytes. Nature 391, 397–401 (1998). 9450757 10.1038/34929

[b28] Apps R. . HIV-1 Vpu Mediates HLA-C Downregulation. Cell Host & Microbe 19, 686–695 (2016). 27173934 10.1016/j.chom.2016.04.005 PMC4904791

[b29] Grabar S. . Prevalence and comparative characteristics of long-term nonprogressors and HIV controller patients in the french hospital database on HIV. AIDS 23, 1163–1169 (2009). 19444075 10.1097/QAD.0b013e32832b44c8

[b30] McLaren P. J. & Carrington M. The impact of host genetic variation on infection with HIV-1. Nat. Immunol. 16, 577–583 (2015). 25988890 10.1038/ni.3147 PMC6296468

[b31] Biassoni R. . Role of amino acid position 70 in the binding affinity of p50.1 and p58.1 receptors for HLA-Cw4 molecules. Eur J Immunol. 27, 3095–3099 (1997). 9464792 10.1002/eji.1830271203

[b32] Moesta A. K. . Humans differ from other hominids in lacking an activating NK cell receptor that recognizes the C1 epitope of MHC class I. J. Immunol. 185, 4233–4237 (2010). 20802150 10.4049/jimmunol.1001951 PMC3124310

[b33] O’Connor G. M. . Peptide-Dependent Recognition of HLA-B*57:01 by KIR3DS1 J. Virol. 89, 5213–5221 (2015). 25740999 10.1128/JVI.03586-14 PMC4442525

[b34] Hilton H. G. . Mutation at Positively Selected Positions in the Binding Site for HLA-C Shows That KIR2DL1 Is a More Refined but Less Adaptable NK Cell Receptor Than KIR2DL3. J. Immunol. 189, 1418–1430 (2012). 22772445 10.4049/jimmunol.1100431 PMC3439511

[b35] Gonzalez-Galarza F. F. . Allele frequency net 2015 update. New features for HLA epitopes, KIR and Disease and HLA adverse drug reaction associations. Nucleic Acid Research 43, D784–788 (2015). 10.1093/nar/gku1166 PMC4383964 25414323

[b36] Marras F. . NK cells in HIV controllers patients express an activated effector phenotype and do not upregulate NKp44 on IL-2 stimulation. Proc. Natl. Acad. Sci. USA 110, 11970–11975 (2013). 23818644 10.1073/pnas.1302090110 PMC3718138

[b37] Kulkarni S., Martin M. P. & Carrington M. KIR genotyping by multiplex PCR-SSP. Methods Mol. Biol. 612, 365–375 (2010). 20033654 10.1007/978-1-60761-362-6_25 PMC3464904

[b38] Biassoni R. . An improved method for HLA-B and -C supratyping. J. Immunol. Methods 426, 29–34 (2015). 26232127 10.1016/j.jim.2015.07.008

[b39] Ugolotti E. . Human leukocyte antigen-B (-Bw6/-Bw4-I80, -T80) and human leukocyte antigen-C (-C1/-C2) subgrouping using pyrosequence analysis. Hum. Immunol. 72, 859–868 (2011). 21664941 10.1016/j.humimm.2011.05.007

[b40] Vanni I., Ugolotti E., Larghero P. & Biassoni R. HLA-B and HLA-C supratyping by pyrosequencing. Meth. Mol. Biol. 1315, 133–151 (2015). 10.1007/978-1-4939-2715-9_11 26103897

[b41] Moroni M. . Spontaneous Control of HIV-1 Viremia in a Subject with Protective HLA-B plus HLA-C Alleles and HLA-C Associated Single Nucleotide Polymorphisms. J. Transl Med. 12, 335–343 (2014). 25477316 10.1186/s12967-014-0335-6 PMC4272524

[b42] Falco M. . Combined genotypic and phenotypic killer cell Ig-like receptor analyses reveal KIR2DL3 alleles displaying unexpected monoclonal antibody reactivity: identification of the amino acid residues critical for staining J. Immunol. 185, 433–441 (2010). 20525888 10.4049/jimmunol.0903632

[b43] Czaja K. . A comprehensive analysis of the binding of anti-KIR antibodies to activating KIRs. Genes Immun. 15, 33–37 (2014). 24173145 10.1038/gene.2013.58

[b44] Greenacre M. Correspondence analysis in practice. (Ed 2nd Boca Raton FL) (Chapman & Hall/CRC 2007).

[b45] https://bitbucket.org/statgen/plink.

