# Supplementary material for: Activating Killer Immunoglobulin Receptors and HLA-C: a successful combination providing HIV-1 control
Source: Sci Rep. 2017 Feb 13;7:42470. doi: 10.1038/srep42470 (PMC5304173; doi:10.1038/srep42470)
Supplement: Supplementary Materials [file srep42470-s1.pdf]

Submitted to Scientific Reports

Activating Killer Immunoglobulin Receptors and HLA-C: a successful combination providing HIV-1 control.

Mauro S. Malnati[1], Elisabetta Ugolotti[2], Maria Cristina. Monti[3], Davide De Battista[1,‡], Irene Vanni[2,†], Domenico Bordo[4], Francesca Sironi[1], Patrizia Larghero[2], Eddi Di Marco[2], Priscilla Biswas[1], Guido Poli[5], Elisa Vicenzi[6], Agostino Riva[7], Maciej Tarkowski[7], Giuseppe Tambussi[8], Silvia Nozza[8], Gino Tripodi[2], Francesco Marras[2], Andrea De Maria[4,9], Angela Pistorio[2], Roberto Biassoni[2]*.

Supplementary Figures

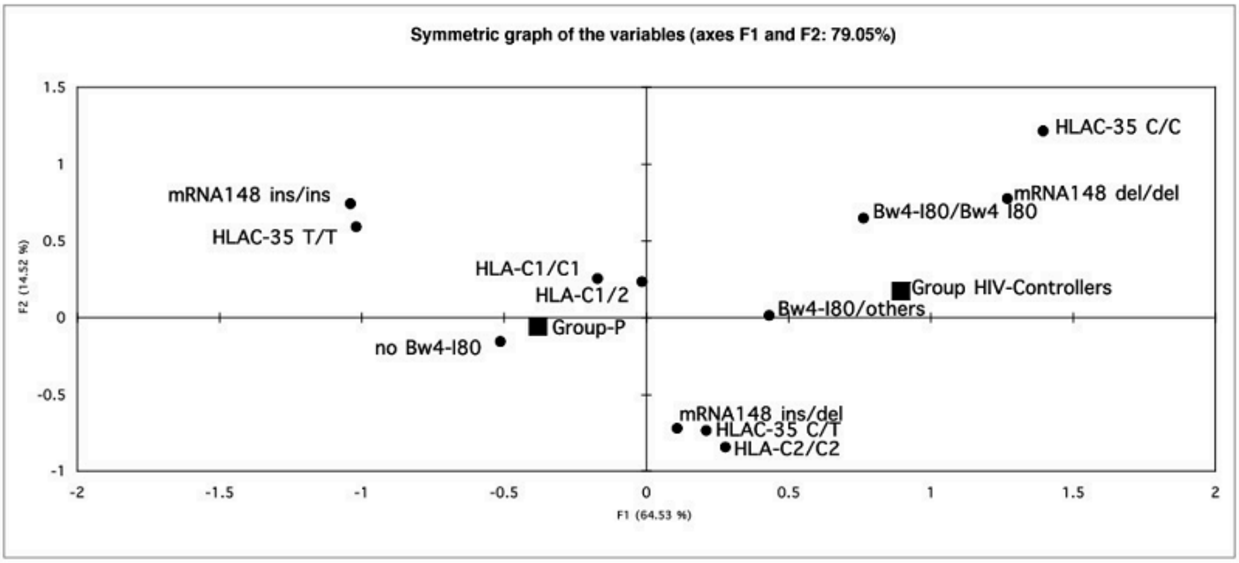

Supplemental Figure 1: Principal component analysis of genetic markers mapping on chromosome 6.

Groups of HIV progressors (Group-P) or controllers (Group HIV-controllers) are indicated with a black filled box.HLA-C, HLA-Bw4I80, -35 SNPs and miRNA148a SNPs homozygous and heterozygous genotypes are shown as black filled circles.

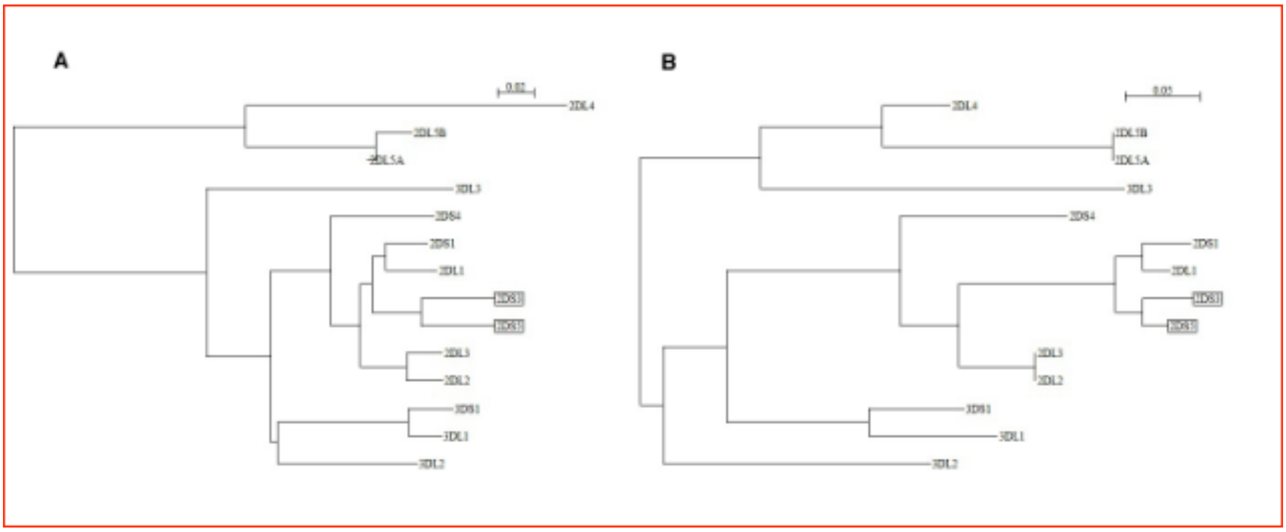

Supplemental Figure 2. Neighbor-joining (N-J) trees of KIR family members.

A: N-J tree obtained from the multiple alignment of KIR sequences obtained from ClustalW; B: N-J tree obtained from the multiple alignment of the residues putatively involved in the interaction with HLA.

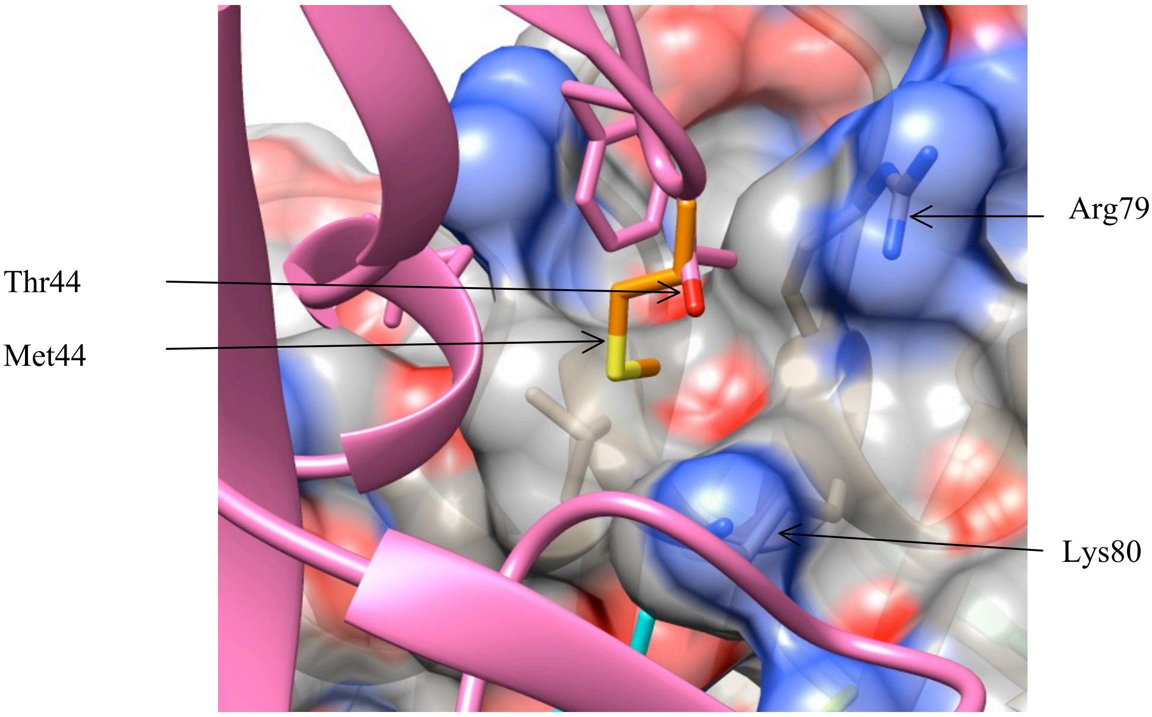

Supplemental Figure 3. Representation of the region involving the KIR:HLA interface.

The modeled KIR2DS5 is shown as ribbon diagram in purple on the upper part of the image, the molecular surface of the MHC protein is shown on the lower part with the positively and negatively charged regions colored in blue (R79 and K80) and red, respectively. The KIR2DL1:Met44 from the crystal structure (PDB, 1im9) and the modeled KIR2DS5:Thr44 are shown in orange and pink, respectively, with the side chain sulfur and oxygen atoms represented in yellow and red, respectively.

Table s1. Clinical, risk factors and demographic characteristics of patients and controls at study entry.

	EC (No.=40)	LTNP (No.=39)	HIV controller (EC+LTNP) (No.=79)	HIV$^+$ART naive (No.=96)	HIV$^+$ART treated (No.=93)	Progressors (HIV$^+$ ART naïve HIV$^+$ ART treated) (No.=189)	Blood-donors (No.=111)
HIV RNA copies/mL (median)	-	1.012	*	55	-	^	-
IQR [1st–3rd]	-	[495–6,481]	*	[9,148–210,000]	-	^	-
CD4 counts (median)	818	716	802	394	593	489	ND
IQR [1st–3rd]	[678–1,099]	[588–996]	[619–1,012]	[269–533]	[482–770]	[348–646]	ND
Heterosexual (%)	11/38 (28.9)	11/39 (28.2)	22/77 (28.6)	46/88 (59.3)	27/85 (31.7)	73/173 (42.2)	ND
MSM/Bisexual (%)	11/38 (28.9)	8/39 (20.5)	19/77 (24.7)	41/88 (37.3)	40/85 (44.7)	81/173 (46.8)	ND
Ex DU (%)	15/38 (39.5)	18/39 (46.2)	33/77 (42.9)	1/88 (1.7)	17/85 (20.0)	18/173 (10.4)	ND
Transfused (%)	1/38 (2.6)	2/39 (5.1)	3/77 (3.9)	-	1/85 (1.2)	1/173 (0.6)	ND
Gender: male number (%)	26/40 (65.0)	23/38 (60.5)	49/78 (62.8)	87/95 (91.6)	75/92 (81.5)	162/187 (86.6)	71/111 (64.0)
Age at blood withdraw (median)	46.0	43.0	44.4	36	46.0	41.5	43.0
IQR [1st–3rd]	[41.4–50.7]	[40.3–47.3]	[40.8–48.4]	[31.6–44.4]	[41.0–52.1]	[34.8–49.0]	[31.9–49.0]

Figures in round parentheses represent percentages; figures in squared parentheses represent fist and third quartiles [1st–3rd q].

EC: Elite Controllers; LTNP: Long Term Non Progressor.

* median and IQR have not been calculated since all EC samples have HIV RNA copies/ml below the limit of detection.

^ median and IQR have not been calculated since all HIV$^+$ ART treated patients have HIV RNA copies/ml below the limit of detection.

Table s2: Linkage disequilibrium analysis

Markers Combination	Linkage Disequilibrium R^2	Linkage Disequilibrium *D'*
rs67384697 G/del vs C1/C2	0.032	0.18
rs9264942 T/C vs C1/C2	0	0.011
rs9264942 T/C vs Bw4–I^{80}	0.05	0.286
rs67384697 G/del vs rs9264942 T/C	0.333	0.621
C1/C2 vs Bw4–I^{80}	0.002	0.065
rs67384697 G/del vs Bw4–I^{80}	0.053	0.319

Supplementary Materials

Sequence alignment and family tree.

The sequence similarity among members of the KIR family was analyzed with the program ClustalW (1) that produced the multiple alignment and the corresponding neighbor-joining (N-J) tree. In the model of the complex HLA:KIR2DS5 the amino acid of the KIR2DS5 molecule in interaction (either hydrogen bond or van der Waals interaction) with HLA residues were assumed to be those aligned with the residues of the KIR2DL1 of the crystal structure of HLA-Cw4:KIR2DL1 (2) (PDB, 1im9) in interaction with the HLA molecule. In the latter complex these interface amino acids were identified according to the program LigPlot (3) and available from the PdbSum web served (4). The amino acid positions are fourteen in number, and on the KIR2DL1 involve positions 44, 45, 68, 71, 72, 105, 106, 132, 133, 135, 181, 183, 184 and 187. Additional four KIR interface residues (positions 21, 70, 73 and 104) not observed in the HLA-Cw4:KIR2DL1 but present in the crystal structure of the closely related HLA-Cw3:KIR2DL2 complex (5) (PDB, 1efx) were included in the list of putative interface KIR residues. Notably the HLA amino acids involved in intermolecular interaction with the bound KIR protein are identical in HLA-Cw4 and HLA-Cw3 (not shown).

Under the assumption that the tertiary structure of the KIR proteins as well as the quaternary structure of the HLA:KIR complex is conserved, it is possible to compare, for each KIR protein, the amino acid composition of the surface patch on the KIR molecule forming the interface with the HLA molecule. The mutual similarity of these patches is summarized by the N-J tree deduced from the alignment of the 14 KIR proteins encompassing the 18 interface residues (Supplemental Figure 2).

Structural analyses.

The three-dimensional model of the immunoglobulin-like domains of KIR2DS5 has been obtained

from the ModBase database (6) (http://modbase.compbio.ucsf.edu/modbase-cgi/index.cgi) based on the crystal structure of the human natural killer cell inhibitory receptor KIR2DL1 (7) (PDB entry, 1nkr). The modeled KIR2DS5 segment spans amino acid positions 27-221 and includes the two Ig-like domains. The sequence identity between KIR2DS5 and template KIR2DL1 along this stretch is 93%, which makes the predicted main chain conformation of the KIR2DS5 highly reliable. However, the side chain conformation of surface residues may still be subject to conformational variability. In order to analyze the role of interface residues in the HLA:KIR2DS5 complex formation, the putative position of the HLA molecule with respect to KIR2DS5 was inferred from the crystal structure of the KIR2DL1 bound to HLA-Cw4 (2) (PDB, 1im9) after optimal structural superimposition of the modeled KIR2DS5 on KIR2DL1. Molecular manipulations were carried out with the program suite Chimera (8) (Supplemental Figure 3).

1. Thompson, J.D., Higgins, D.G., Gibson, T.J. CLUSTAL W: improving the sensitivity of progressive multiple sequence alignment through sequence weighting, position-specific gap penalties and weight matrix choice. *Nucleic Acid Res.* **22**, 4673–4680 (1994).
2. Fan, Q.R., Long, E., Wiley, D.C. Cristal structure of the human Natural Killer cell inhibitory receptor KIR2DL1-HLA-Cw4 complex. *Nat. Immunol.* **2**, 452–60 (2001).
3. Wallace, A.C., Laskowski, R.A., Thornton, J.M. LIGPLOT: a program to generate schematic diagrams of protein-ligand interactions. *Protein Eng.* **8**, 127–134 (1995).
4. Laskowski, R.A. PDBsum: summaries and analyses of PDB structures. *Nucleic Acids Res.* **29**, 221–222 (2001).
5. Boyington, J.C., Motyka, S.A., Schuck, P., Brooks, A.G., Sun, P.D. Crystal structure of an NK cell immunoglobulin-like receptor in complex with its class I MHC ligand. *Nature* **405**, 537–43 (2000).
6. Pieper, U., et al. ModBase, a database of annotated comparative protein structure models and associated resources. *Nucleic Acids Res.* **42**, D336–346 (2014).

7. Fan, Q.R., et al. Structure of the inhibitory receptor for human natural killer cells resembles haematopoietic receptors. *Nature* **389**, 96–100 (1997).
8. Pettersen, E.F., et al. UCSF Chimera--a visualization system for exploratory research and analysis. *J. Comput. Chem.* **25**, 1605–1612 (2004).
